# Zinc, Copper, and Calcium: A Triangle in the Synapse for the Pathogenesis of Vascular-Type Senile Dementia

**DOI:** 10.3390/biom14070773

**Published:** 2024-06-28

**Authors:** Masahiro Kawahara, Ken-ichiro Tanaka, Midori Kato-Negishi

**Affiliations:** Department of Bio-Analytical Chemistry, Research Institute of Pharmaceutical Sciences, Faculty of Pharmacy, Musashino University, 1-1-20 Shinmachi, Nishitokyo-shi 202-8585, Tokyo, Japan

**Keywords:** ischemia, calcium homeostasis, endoplasmic reticulum stress, reactive oxygen species (ROS)

## Abstract

Zinc (Zn) and copper (Cu) are essential for normal brain functions. In particular, Zn and Cu are released to synaptic clefts during neuronal excitation. Synaptic Zn and Cu regulate neuronal excitability, maintain calcium (Ca) homeostasis, and play central roles in memory formation. However, in pathological conditions such as transient global ischemia, excess Zn is secreted to synaptic clefts, which causes neuronal death and can eventually trigger the pathogenesis of a vascular type of senile dementia. We have previously investigated the characteristics of Zn-induced neurotoxicity and have demonstrated that low concentrations of Cu can exacerbate Zn neurotoxicity. Furthermore, during our pharmacological approaches to clarify the molecular pathways of Cu-enhanced Zn-induced neurotoxicity, we have revealed the involvement of Ca homeostasis disruption. In the present review, we discuss the roles of Zn and Cu in the synapse, as well as the crosstalk between Zn, Cu, and Ca, which our study along with other recent studies suggest may underlie the pathogenesis of vascular-type senile dementia.

## 1. Introduction

Both zinc (Zn) and copper (Cu) are essential elements for most organisms and play critical roles in normal brain function. Moreover, Zn is stored in presynaptic vesicles of glutamatergic neurons and is secreted from these vesicles into synaptic clefts alongside glutamate during neuronal excitation [1]. Secreted Zn acts as an endogenous neuromodulator for information processing; it contributes to synaptic plasticity and memory formation [2]. Cu is also secreted to synaptic clefts and modulates neuronal information, similar to Zn [3]. Thus, both secreted Zn and Cu regulate overall neuronal excitability; they also regulate calcium (Ca) homeostasis under normal physiological conditions.

However, despite their importance, excess Zn and Cu are neurotoxic. Increasing evidence suggests that dyshomeostasis of these metals is involved in the pathogenesis of various neurodegenerative diseases including Alzheimer’s disease, prion diseases, Parkinson’s disease, dementia with Lewy bodies, amyotrophic lateral sclerosis, Wilson disease, and Menkes disease [4,5,6,7,8]. In particular, it is widely accepted that excess Zn, which is secreted to the synapses under conditions of transient global ischemia, causes neurodegeneration and may trigger the pathogenesis of vascular-type senile dementia (VTD) [9,10,11].

To better understand the pathogenesis of VTD, we have investigated the molecular pathways of Zn-induced neuronal death [12,13,14,15,16,17]. We have also attempted to identify protective substances against Zn-induced neurotoxicity as possible treatments for VTD [18,19]. Our results indicate that the endoplasmic reticulum (ER) stress pathway is implicated in Zn-induced neurodegenerative processes [15,16]. Furthermore, we found that sublethal concentrations of Cu exacerbate Zn-induced neuronal death [20]. It is therefore possible that the colocalization of Cu and Zn in the same synapse during ischemia disrupts Ca homeostasis and triggers neuronal death. In the present article, we review the physiological and pathophysiological roles of Zn and Cu in the synapse and discuss the involvement of Ca homeostasis and other neurodegenerative processes in Cu-enhanced Zn-induced neurotoxicity, as well as their link to the pathogenesis of VTD.

## 2. Roles of Zn and Cu in the Brain

Zn is the second most abundant essential element in the body. The human body contains approximately 2 g of Zn, which is mainly distributed in the blood, kidney, liver, bone, and brain. Zn acts as a cofactor for more than 300 enzymes or metalloproteins and is involved in important biological functions such as mitotic cell division, cell growth, immune responses, protein synthesis, and DNA/RNA synthesis [21]. Thus, Zn deficiency causes various adverse symptoms such as impaired immune responses, skin disorders, and growth retardation [22]. Moreover, increasing evidence suggests that Zn acts as a signaling molecule, similar to Ca [23]. Zn signaling pathways have been implicated in various biological functions, including immune responses, fertilization, and cell division.

Zn signaling is also observed in the brain. Zn is present at the highest concentrations in the hippocampus, amygdala, cerebral cortex, thalamus, and olfactory cortex. Although most Zn is strongly bound to metalloproteins or enzymes, a relatively large amount of Zn (approximately 10% or more) exists in the presynaptic vesicles of excitatory glutamate neurons either as free Zn ions (Zn^2+^) or in loosely bound forms. During neuronal excitation, synaptic Zn is secreted from presynaptic vesicles into synaptic clefts alongside glutamate [2]. This secreted Zn reportedly binds to *N*-methyl-D-aspartate (NMDA)-type glutamate receptors (NMDA-R) and inhibits their functions [24]. Morabito et al. reported that secreted Zn modulates dendritic functions via NMDA-R in an activity-dependent manner [25]. Zn also binds to other receptors, including amino-3-hydroxy-5-methyl-4-isoxazolepropionic acid (AMPA)-type glutamate receptors (AMPA-R), γ-amino butyric acid (GABA) receptors, glycine receptors, and P2-type purinergic receptors [26,27]. Moreover, Zhang et al. demonstrated the appearance of spontaneous and synchronous changes in intracellular Zn^2+^ levels ([Zn^2+^]_i_) (namely, Zn spikes) in cultured hippocampal neurons [28]. The Zn spikes are correlated with similar oscillatory changes in intracellular Ca^2+^ levels ([Ca^2+^]_i_) (namely, Ca^2+^ spikes), which regulate synapse maturation and neuronal differentiation [29]. Thus, synaptic Zn acts as an endogenous neuromodulator that provides spatiotemporal information regarding neuronal plasticity and is essential for memory formation. Indeed, hippocampal Zn is essential for the induction of long-term potentiation (LTP), a form of synaptic information storage that has become a well-known paradigm for the mechanisms underlying memory formation [30]. Consistent with these findings, Zn deficiency in childhood causes delayed mental development and learning disabilities [31]. In adults, Zn deficiency causes odor disorders, taste disorders, fear-conditioning disorders, and related emotional activity [21]. Furthermore, Zn deficiency can cause seizures, epilepsy, or convulsions. Serum Zn levels are reportedly lower in children with febrile seizures [32].

Cu is the third most abundant essential element in the body. In the brain, Cu is localized in the thalamus, substantia nigra, striatum, and hippocampus. Cu acts as a cofactor of various enzymes, including cytochrome C, lysyl oxidase, uricase, dopamine hydroxylase, and tyrosinase [33]. It also plays a central role in neurotransmitter synthesis and myelination. Cu is also involved in iron (Fe) homeostasis because ceruloplasmin, a Cu-binding protein, acts as a ferroxidase that converts Fe^2+^ to Fe^3+^. Additionally, both Cu and Zn are neuroprotective against reactive oxygen species (ROS) because both elements are components of Cu/Zn superoxide dismutase. Similar to Zn, Cu is localized in the synapse and can be secreted into synaptic clefts during neuronal firing [3,34]. Secreted Cu regulates neuronal excitability by binding to NMDA-R and AMPA-R.

Zn and Cu are suggested to modulate neuronal excitability in a dose-dependent biphasic manner [35]. Delgado et al. revealed the biphasic functions of Zn and Cu, reporting that neuronal firing rates increase after exposure to nanomolar levels of Zn and Cu but decrease after exposure to micromolar concentrations [36]. The levels of Zn and Cu in the synapse are therefore critical. Notably, cerebrospinal fluid (CSF) concentrations of Cu and Zn in healthy individuals are less than 1 µM [37]. However, the synaptic cleft is a small compartment that is conceptualized as a cylinder with a 120 nm radius and height of 20 nm, and the total volume of synaptic clefts is estimated to account for approximately 1% of the extracellular space of the brain, which is similar to the CSF volume [38]. It is thus possible that the Cu and Zn concentrations of synapses are much higher than those of the CSF. Although the synaptic levels of Zn and Cu remain controversial, Vogt et al. estimated that Zn concentrations in synapses are 1–100 μM [39]. Kardos et al. reported that approximately 100 μM Cu is released into the synaptic cleft using atomic absorption spectroscopy [40]. By contrast, a study using a Cu-sensitive fluorescent probe demonstrated that approximately 3 μM Cu is released into the synaptic cleft [41].

Despite their abundance in the synapse, excess Zn and Cu are neurotoxic. In particular, excess Cu produces ROS and is highly neurotoxic because Cu is a redox-active metal that exists as oxidized Cu^2+^ and reduced Cu^+^. Therefore, the concentrations of both Zn and Cu need to be precisely controlled. Zn homeostasis is regulated by metallothioneins (MTs) and Zn transporters. MTs possess seven binding sites for Zn, Cu, cadmium (Cd), and other metals, and regulate the detoxification of these elements. Although MT-1 and MT-2 are ubiquitously present in the body, MT-3 is predominantly present in neurons or glia. There are two types of Zn transporters: ZnT transporter and Zrt/Irt-like protein (ZIP) transporter [42]. ZnT transporters export Zn from the cytosol to the extracellular space, whereas ZIP transporters control Zn influx into cells or subcellular organelles. Of the 10 ZnT transporters in mammals, ZnT-3 is central to the accumulation of Zn in synaptic vesicles. Moreover, ZnT-1 is widely expressed throughout the whole body; it plays a pivotal role in Zn efflux and protects against Zn toxicity. Elevated Zn causes ZnT-1 upregulation and the translocation of ZnT-1 onto the plasma membrane [43]. Interestingly, ZnT-1 possesses other neuronal functions in addition to Zn removal. ZnT-1 is reportedly localized in postsynaptic membranes, where it binds with NMDA-R and regulates the functions [44]. ZnT-1 also binds to L-type voltage-gated Ca^2+^ channels (L-VGCC) and inhibits their functions [45]. ZnT-1 also activates T-type Ca^2+^ channels [46]. Meanwhile, of the 14 ZIP transporters in mammals, ZIP4 predominantly regulates intracellular Zn influx in the brain. ZIP4 is reportedly present in both neurons and synapses [47].

Cu homeostasis is controlled by Cu transporters such as ATPase copper transporting alpha (ATP7A), ATPase copper transporting beta (ATP7B), and copper transporter 1 (CTR1) [33]. Genetic disorders of ATP7B cause excess Cu and trigger the pathogenesis of Wilson disease, whereas genetic disorders of ATP7A cause Cu deficiency in Menkes disease. ATP7A and ATP7B are predominantly localized in the Golgi apparatus [48], and ATP7A has been implicated in axonal transport and synaptogenesis [49]. CTR1, which regulates Cu^+^ influx, is reportedly localized in the synapse [50].

Recent studies have suggested that several neurodegenerative disease-related proteins are localized in synapses and regulate metal homeostasis [51]. The accumulation of β-amyloid protein and its associated neurotoxicity is believed to play a critical role in the pathogenesis of Alzheimer’s disease. Its precursor protein, amyloid precursor protein (APP), can bind to Zn, Cu, and Fe and regulate metal homeostasis. APP also has the ability to reduce Cu^2+^ to Cu^+^ [52]. In addition, the conformational conversion of normal cellular prion protein (PrP^C^) to its pathogenic form (PrP^SC^) underlies the pathogenesis of prion diseases such as Creutzfeldt–Jakob disease, scrapie, and bovine spongiform encephalopathy. PrP^C^ is a metal-binding protein that binds to Cu, Zn, and Fe and is implicated in Cu and Fe influx [53]. Interestingly, there is an evolutional similarity between PrP^C^ and ZIP sequences [54]. PrP^C^ is localized in postsynaptic membranes, where it binds with AMPA-R, acts as a Zn sensor in the synapse, and regulates Zn influx [55]. PrP^C^ also regulates the functions of NMDA-R and AMPA-R differently in a Cu-dependent manner [56].

**Figure 1:**
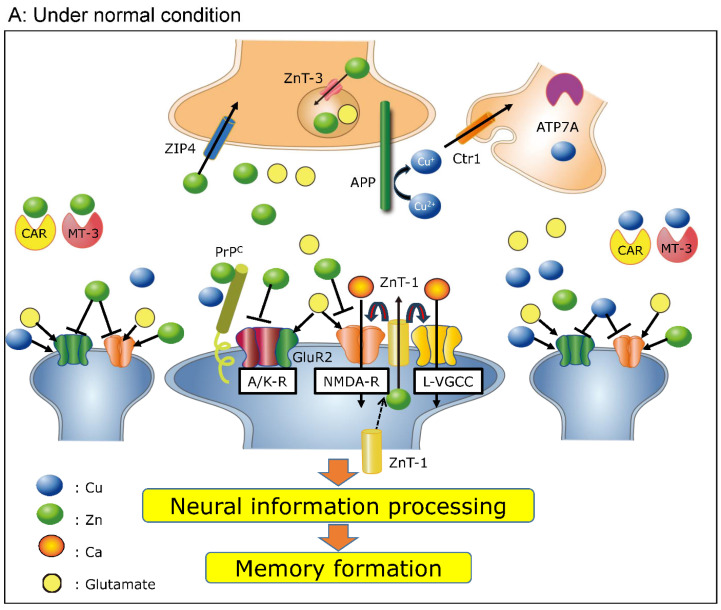

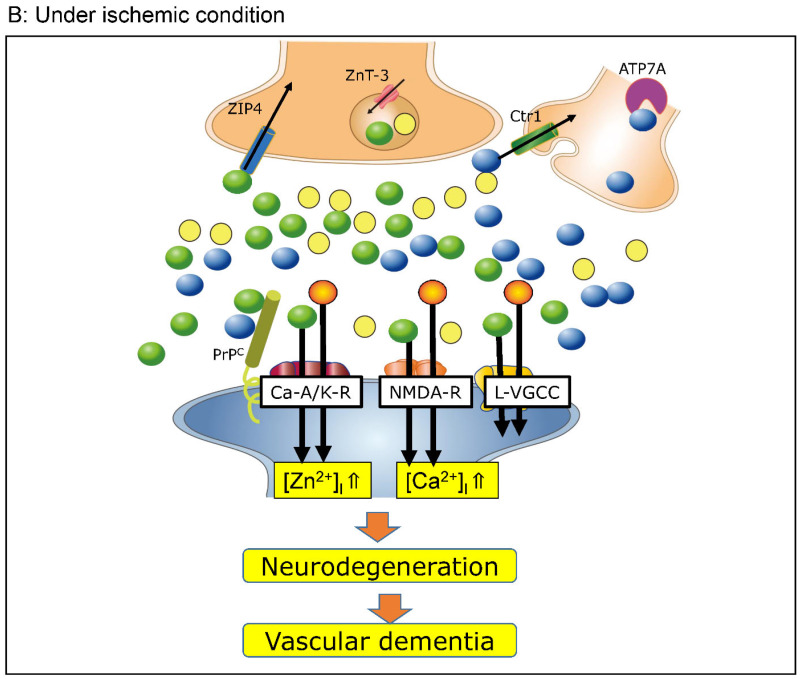
Roles of Zn and Cu in the synapse under physiological and pathophysiological conditions: (**A**) Roles of Zn and Cu under normal physiological conditions. Zn and Cu are secreted from presynaptic vesicles to the synaptic clefts with glutamate during neuronal firing. Secreted Zn binds to *N*-methyl-d-aspartic acid-type glutamate receptors (NMDA-R) and amino-3-hydroxy-5-methyl-4-isoxazolepropionic acid (AMPA)/kainite type glutamate receptors (A/K-R) and inhibits their functions. Zn enhances the expression of the zinc transporter ZnT-1 or the translocation of ZnT-1 to synaptic membranes. Thereafter, ZnT-1 binds to NMDA-R and L-type voltage-gated Ca^2+^ channels (L-VGCC) to regulate overall brain excitability and maintain Ca homeostasis. Secreted Cu regulates NMDA-R and A/K-R by binding with normal cellular prion protein (PrP^C^). PrP^C^ also regulates Zn^2+^ influx by binding with A/K-R. Secreted Zn and Cu spill over to neighboring synapses and inhibit or potentiate neuronal excitability in a biphasic dose-dependent manner. Finally, Zn and Cu contribute to information processing and memory formation. ZIP-4 can reuptake secreted Zn, and CTR1 can reuptake Cu once it has been reduced to Cu^+^ by amyloid precursor protein (APP). The subtle balance of Zn, Cu, and Ca maintains synaptic functions. (**B**) Roles of Zn and Cu under pathophysiological conditions. In transient global ischemia conditions, widespread, prolonged excitation occurs in the brain, and excess Zn and Cu can be secreted to synaptic clefts with glutamate. Secreted excess Zn causes the acute reduction of the GluR2 subunit of A/K-R, and Ca^2+^-permeable A/K-R (Ca-A/K-R) appears. Both Ca^2+^ and Zn^2+^ are intracellularly translocated via Ca-A/K-R, NMDA-R, and L-VGCC. Increased intracellular Ca^2+^ levels ([Ca^2+^]i) and intracellular Zn^2+^ levels ([Zn^2+^]i) then cause various neurodegenerative pathways, ultimately triggering the pathogenesis of vascular-type senile dementia. Colored circles represent Zn, Cu, Ca, and glutamate.

We have used these findings to illustrate the possible roles of secreted Zn and Cu in the synapse under normal physiological conditions (Figure 1A). Zn and/or Cu are secreted to the synaptic cleft during neuronal excitation alongside glutamate. Glutamate, an excitatory neurotransmitter, binds to its receptors and induces Ca influx into postsynaptic neurons. Secreted Zn inhibits NMDA-R, controls over-excitation, and maintains [Ca^2+^]_i_ levels. Furthermore, increased Zn levels induce the upregulation of ZnT-1 and the accumulation of ZnT-1 in synaptic membranes. Thereafter, ZnT-1 controls NMDA-R and L-VGCC to suppress the increase in [Ca^2+^]_i_. Secreted Cu also binds to NMDA-R and inhibits its function. Moreover, Cu binds to PrP^C^ and differently regulates NMDA-R and AMPA-R. It is also possible that MT-3 [57] and carnosine (β-alanyl histidine) [58] bind to these metals and regulate Zn and Cu homeostasis in the synapse.

Both glutamate and Zn (or Cu) are simultaneously secreted to synaptic clefts and diffusely spill over to neighboring synapses and thereafter transfer information about neuronal firing [59]. This spillover of Zn and Cu can then inhibit or activate neuronal firing in a biphasic manner, depending on their concentrations. It is therefore possible that Zn and Cu collaborate in the synapse to regulate overall excitability and maintain Ca levels. Secreted Zn and Cu can be rapidly removed from the synapse. ZIP4 reuptakes secreted Zn, which is then transferred to presynaptic vesicles via ZnT-3. Similarly, CTR1 reuptakes secreted Cu once it has been reduced to Cu^+^ by APP. It is therefore unlikely that excessive concentrations of Zn and Cu coexist in the same synapse under normal conditions.

## 3. Zn and Cu under Pathological Conditions

Excess Zn is believed to play a central role in neurodegeneration after transient global ischemia and ultimately in the pathogenesis of VTD [60,61]. The incidence of senile-type dementia increases with age and has been estimated to affect more than seven million people in Japan in 2025, a number that is increasing annually. VTD is responsible for approximately one-third of all cases of senile-type dementia. VTD is a degenerative cerebrovascular disease; its risk factors include aging, sex (male), diabetes, and high blood pressure [62]. The most common type of VTD is caused by a series of small strokes or ischemia. After transient global ischemia or stroke, the interruption of blood flow and resulting oxygen–glucose deprivation occurs, thus inducing long-lasting membrane depolarization over wide regions of the brain [61]. Thereafter, the excessive release of glutamate into synaptic clefts causes the overstimulation of its receptors and the subsequent entry of large quantities of Ca^2+^ into glutamate-responsive neurons. This then triggers the delayed death of vulnerable populations of neurons in the hippocampus or cerebral cortex. The development of an infarct and subsequent cognitive dysfunction mark the pathogenesis of VTD in older adults. Notably, approximately 30% of stroke patients show symptoms of dementia within 3 months of the initial stroke [63].

In 1996, Koh et al. reported that Zn accumulates within the cell bodies of degenerating neurons following transient global ischemia [64]. The administration of Ca ethylenediaminetetraacetic acid (Ca EDTA), a membrane-impermeable Zn chelator, protects hippocampal neurons after transient global ischemia and reduces infarct volumes [65]. Under ischemic conditions, increased [Zn^2+^]_i_ (namely, Zn translocation) occurs in vulnerable neurons in the CA1 or CA3 hippocampal regions prior to the onset of delayed neuronal death after transient global ischemia. This Zn translocation is the primary event in the pathway of Zn-induced neuronal death. Sensi et al. used a Zn-sensitive fluorescent dye to reveal the three major routes of Zn translocation in cultured cortical neurons: L-VGCC, NMDA-R, and Ca^2+^-permeable AMPA/kainite-type glutamate receptors (Ca-A/K-R) [66]. Under normal physiological conditions, most hippocampal neurons express A/K-R, which are composed of four subunits (GluR1 to GluR4) and are poorly permeable to Ca^2+^ and Zn^2+^. However, under ischemic conditions, there is an acute reduction in GluR2 subunit expression in the hippocampus, and AMPA-R that lack GluR2 subunits become more permeable to Ca^2+^ and Zn^2+^ in vulnerable neurons [67]. Thereafter, the appearance of Ca-A/K channels causes increased permeability of Ca^2+^ and enhances its toxicity. The permeability of Ca^2+^ through Ca-A/K-R is greater than that through NMDA-R channels or L-VGCC. Therefore, Ca-A/K channel expression—and the entry of Ca^2+^ and Zn^2+^ through these channels—is a mediator of delayed neuronal death after ischemia. Zn has also been implicated in the transcriptional regulation of Ca-A/K channels, and Ca EDTA can attenuate the ischemia-induced downregulation of the gene encoding GluR2 [65]. These interactions of Zn and Cu in the pathological synapse are illustrated in Figure 1B.

Under transient global ischemia conditions, there is widespread, long-lasting neuronal excitation in the brain. This results in the coexistence of both excess Cu and excess Zn in the same synapses, although this is rare in normal physiological conditions by the rapid removal from the synapse. Given that Cu enhances Zn-induced neuronal death [20], it is possible that Zn and Cu may collaborate to cause neuronal death, and ultimately VTD pathogenesis, under ischemic conditions. Consistent with this idea, serum Cu is reportedly associated with ischemic stroke [68,69,70].

## 4. Characteristics of Zn-Induced Neurotoxicity and Cu-Enhanced Zn Neurotoxicity

Understanding the molecular mechanisms of Zn-induced neuronal death is crucial considering the importance of Zn in VTD pathogenesis. To this end, we used an in vitro model system using GT1-7 cells (an immortalized hypothalamic neuronal cell line). GT1-7 cells were originally developed by Mellon et al. by genetically targeting the tumorigenesis of mouse hypothalamic neurons [71]. These cells possess neuronal characteristics such as neurite extension, gonadotropin-releasing hormone (GnRH) secretion, and the expression of neuron-specific proteins or receptors (including microtubule-associated protein 2, tau protein, neurofilament, synaptophysin, GABAA receptors, dopamine receptors, and L-type Ca^2+^ channels). However, excess glutamate does not cause GT1-7 cell death because these cells lack or have low expression of glutamate receptors [58]. By contrast, we found that Zn causes GT1-7 cell death in a dose- and time-dependent manner [12] and noted that degenerated GT1-7 cells exhibit terminal deoxynucleotidyl transferase-mediated biotinylated dUTP nick-end labeling (TUNEL) and DNA fragmentation. Moreover, GT1-7 cells are very sensitive to Zn compared with other neuronal cells, including primary cultured hippocampal neurons and PC12 cells [14]. These properties make the GT1-7 cell line an excellent model system for investigating Zn-induced neurotoxicity.

**Figure 2:**
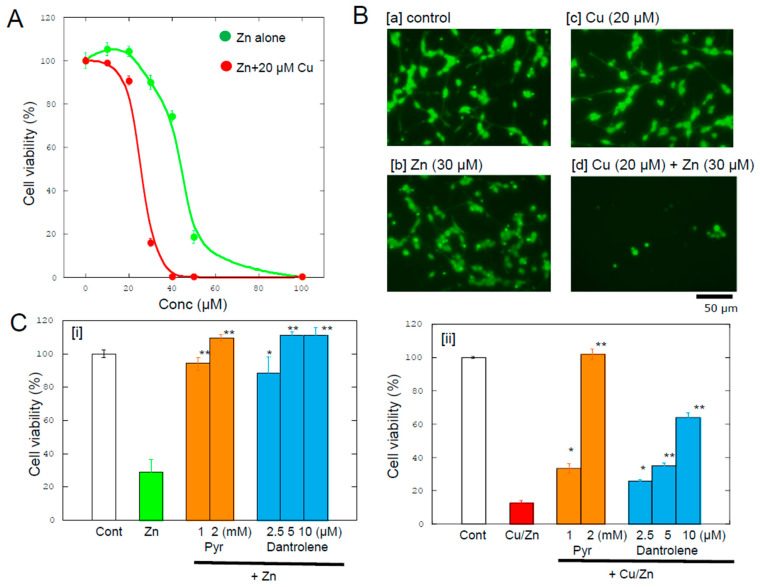
The coexistence of Cu exacerbates Zn-induced neurotoxicity: (**A**) Various concentrations of ZnCl_2_ were applied to GT1-7 cells with or without 20 µM CuCl_2_. After 24 h of exposure, cell viability was analyzed using a Cell-Titer Glo2 assay. Data are expressed as the mean ± standard error of the mean (SEM), *n* = 6. (**B**) Morphological changes in GT1-7 cells were observed by fluorescent microscopy stained by carboxyfluorescein after 24 h of exposure to [**a**] control, [**b**] 20 µM CuCl_2_, [**c**] 30 µM ZnCl_2_, and [**d**] 20 µM CuCl_2_ with 30 µM ZnCl_2_. (**C**) Effects of sodium pyruvate and dantrolene on Zn neurotoxicity and Cu/Zn neurotoxicity. [**i**]: GT1-7 cells were exposed to 50 µM ZnCl_2_ with or without sodium pyruvate (Pyr; 1~2 mM) and dantrolene (2.5~10 µM). After 24 h, cell viability was analyzed using a Cell-Titer Glo2 assay. Data are expressed as the mean ± SEM, *n* = 6. * *p* < 0.05, ** *p* < 0.01 (vs. Zn alone). [**ii**]: GT1-7 cells were exposed to 20 µM CuCl_2_ and 30 µM ZnCl_2_ with or without sodium pyruvate (Pyr; 1~2 mM) and dantrolene (2.5~10 µM). After 24 h, cell viability was analyzed using a Cell-Titer Glo2 assay. Data are expressed as the mean ± SEM, *n* = 6. * *p* < 0.05, ** *p* < 0.01 (vs. Cu/Zn alone).

We therefore explored the detailed characteristics of Zn-induced death in GT1-7 cells. Our pharmacological results revealed that several substances, including sodium pyruvate and *o*-phenanthroline (a Zn chelator), attenuate the viability of GT1-7 cells after Zn exposure. By contrast, antagonists or agonists of excitatory neurotransmitters (D-APV, glutamate, and CNQX) or inhibitory neurotransmitters (bicuculline, muscimol, baclofen, and GABA) do not attenuate GT1-7 cell viability [14]. We also evaluated the involvement of other metal ions in Zn-induced neurotoxicity and found that the coexistence of Cu^2+^ or nickel ions (Ni^2+^) significantly exacerbates Zn-induced neurotoxicity in GT1-7 cells [20]. Figure 2A exhibits the dose–response curve of cell viability after 24 h of exposure to ZnCl_2_ with or without 20 μM CuCl_2_. Although GT1-7 cells extend neurites under control conditions (Figure 2(Ba)), neurite retraction and degenerated cells can be observed after exposure to 30 μM ZnCl_2_ (Figure 2(Bb)). By contrast, exposure to 20 μM CuCl_2_ does not affect cell viability or morphology (Figure 2(Bc)). However, the coexistence of 20 μM Cu and 30 μM Zn remarkably exacerbates toxicity, and most cells are markedly degenerated (Figure 2(Bd)).

We therefore focused on the molecular pathways of Cu-enhanced Zn-induced neurotoxicity (Cu/Zn neurotoxicity). In a comprehensive DNA microarray analysis, we found that several genes are upregulated after coexposure to Cu and Zn compared with either Zn or Cu alone. Thus, we used reverse transcription polymerase chain reaction (RT-PCR) to investigate the detailed expression of such genes.

After exposure to Zn alone, the expression of metal-related genes (*ZnT1*, *MT1*, and *MT2*), ER stress-related genes (CCAAT-enhancer-binding protein homologous protein [*CHOP*], growth arrest and DNA-damage-inducible gene 34 [*GADD34*], activating transcription factor 4 [*ATF4*], immunoglobulin binding protein [*BIP*], ER degradation-enhancing α-mannosidase-like protein [*EDEM*], spliced X-box binding protein-1 [*sXBP1*], glucose-regulated protein 94 [*GRP94*], and protein disulfide isomerase [*PDI*]), and synaptic plasticity-related genes (activity-related cytoskeleton protein [*Arc*]) are upregulated [15,16]. By contrast, exposure to Cu^2+^ alone does not induce significant changes in any of these genes. Notably, however, cells coexposed to Cu and Zn exhibit a synergistic increase in the expression levels of *ATF4*, *CHOP*, and *GADD34* [20]. These results indicate that the ER stress pathway is involved in Cu/Zn neurotoxicity. The ER stress pathway, which impairs ER function and leads to the accumulation of unfolded or misfolded proteins, has been implicated in many neurodegenerative diseases, including Alzheimer’s disease, Parkinson’s disease, and cerebral ischemia [72]. ER stress is mediated by three sensors embedded in the ER membrane: PKR-like endoplasmic reticulum eIF2a kinase (PERK), inositol requiring enzyme 1 (IRE1), and activating transcription factor 6 (ATF6). Of the three axes of the ER stress pathway, it is possible that the ATF4/CHOP/GADD34 axis is responsible for Cu/Zn neurotoxicity because the genes related to the PERK branch (*ATF4*, *CHOP*, and *GADD34*) are upregulated. In the PERK branch, ATF4 induces CHOP, which then triggers an intrinsic apoptotic pathway, including caspase cascades [73]. Thereafter, CHOP induces GADD34 protein (protein phosphatase 1 regulatory subunit 15A). Dantrolene, an inhibitor of the ER stress pathway, attenuates both Cu/Zn neurotoxicity and Zn-induced neurotoxicity (Figure 2C). Carnosine attenuates Zn-induced neurotoxicity as well as neurotoxicity caused by ER stress inducers, such as thapsigargin and tunicamycin [16].

With the coexposure of Cu and Zn, we also found the upregulation of genes related to the stress-activated protein kinase/c-Jun amino-terminal kinase (SAPK/JNK) signaling pathway [74]. Furthermore, Cu/Zn neurotoxicity is attenuated by SP600125, an inhibitor of the SAPK/JNK signaling pathway. This signaling pathway plays an important role in apoptotic cell death, necroptosis, and autophagy [75].

We have demonstrated that sodium pyruvate attenuates both Cu/Zn neurotoxicity and Zn neurotoxicity, as shown in Figure 2C [76]. We have also reported that pyruvic acid suppresses Zn-induced mitochondrial injury in GT1-7 cells. Pyruvic acid is an important organic acid that is involved in various metabolic pathways, such as glycolysis and the tricarboxylic acid cycle. Pyruvic acid is thought to have a protective effect in neuronal cells because it improves energy levels via cellular oxidized nicotinamide adenine dinucleotide (NAD^+^) and ATP. Notably, pyruvic acid is also reported to attenuate Zn-induced death in cultured cortical neurons [77]. Pyruvic acid also attenuates neuronal death induced by transient global ischemia in vivo [78].

We have also demonstrated that antioxidants such as thioredoxin–human albumin fusion protein (HSA-Trx) and seleno-L-methionine (Se-Met) can attenuate Cu/Zn neurotoxicity [79,80]. Cu is a redox-active metal that can exist as Cu^2+^ or Cu^+^, whereas Zn is a non-redox active metal. While exposure to Cu^2+^ causes an intracellular increase in ROS in GT1-7 cells, exposure to Zn^2+^ does not produce ROS or influence Cu-induced ROS production [79]. Consistent with these findings, we found that HSA-Trx inhibits ROS production and attenuates Cu/Zn neurotoxicity [79]. Moreover, seleno-L-methionine induces glutathione peroxidase and suppresses Cu/Zn neurotoxicity [80].

## 5. Involvement of Ca Homeostasis in Cu/Zn Neurotoxicity

**Figure 3:**
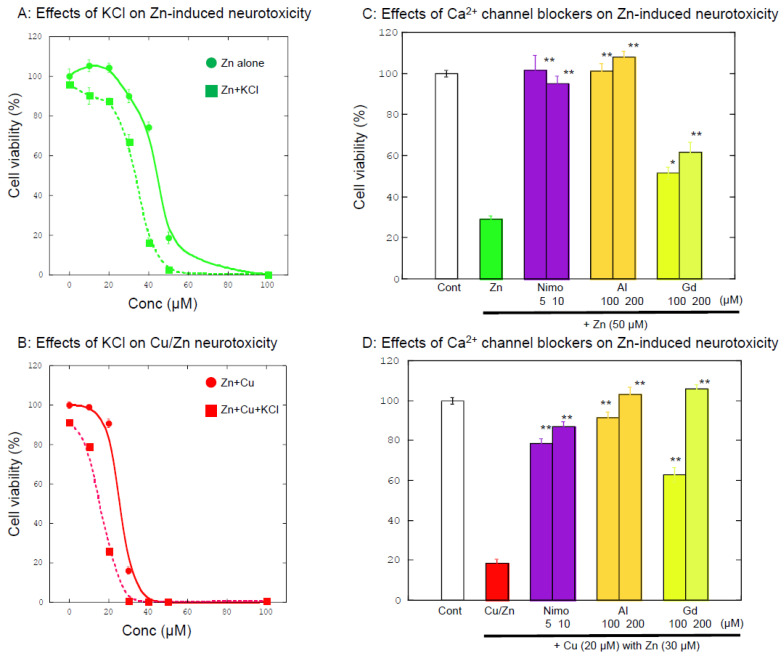
Effects of substances related to Ca influx on Zn neurotoxicity and Cu/Zn neurotoxicity: (**A**) GT1-7 cells were exposed to various concentrations of ZnCl_2_ with or without 150 mM KCl. (**B**) GT1-7 cells were exposed to 20 µM CuCl_2_ with various concentrations of ZnCl_2_, with or without 150 mM KCl. After 24 h, cell viability was analyzed using a Cell-Titer Glo2 assay. Data are expressed as the mean ± SEM, *n* = 6. (**C**) GT1-7 cells were exposed to 50 µM ZnCl_2_ with or without nimodipine (nimo), AlCl_3_, and GdCl_3_. After 24 h, cell viability was analyzed using a Cell-Titer Glo2 assay. Data are expressed as the mean ± SEM, *n* = 6. * *p* < 0.05, ** *p* < 0.01 (vs. Zn). (**D**) GT1-7 cells were exposed to 20 µM CuCl_2_ and 30 µM ZnCl_2_ (Cu/Zn) with or without nimodipine (Nimo), AlCl_3_, and GdCl_3_. After 24 h, cell viability was analyzed using a Cell-Titer Glo2 assay. Data are expressed as the mean ± SEM, *n* = 6. ** *p* < 0.01 (vs. Cu/Zn).

Considering the importance of Ca^2+^ homeostasis in various neurodegenerative pathways, we examined the effects of substances that influence [Ca^2+^]_i_ using GT1-7 cells. The addition of 150 mM KCl, which causes GT1-7 cell depolarization and increases [Ca^2+^]_i_, exacerbates both Zn-induced neurotoxicity and Cu/Zn neurotoxicity (Figure 3A,B). We also found that nimodipine, a blocker of L-type Ca^2+^ channels, attenuates both Cu/Zn neurotoxicity and Zn neurotoxicity (Figure 3C,D). These results are consistent with previous findings that Zn neurotoxicity in PC-12 cells is attenuated by nimodipine and enhanced by S(−)-Bay K 8644 (an L-type Ca^2+^ channel activator) [14,81].

Furthermore, we demonstrated that the addition of aluminum (Al^3+^) ions and gadolinium (Gd^3+^) ions also attenuates both Cu/Zn neurotoxicity and Zn neurotoxicity, as shown in Figure 3C,D. We have already revealed that Zn exposure causes an increase in [Ca^2+^]_i_ in GT1-7 cells using a high-resolution multi-site video imaging system [14]. Detailed analysis of Zn-induced [Ca^2+^]_i_ revealed that the pretreatment of Al^3+^ significantly blocked the Zn-induced [Ca^2+^]_i_ elevations. It is widely known that Al^3+^ inhibits various types of Ca^2+^ channels such as N-, P-, Q-, and L-type Ca^2+^ channels [82,83], and Gd^3+^ is a blocker of non-selective cation channels [84] or voltage-gated Ca^2+^ channels [85]. Although Al is a neurotoxin, Al does not influence cell viability under this experimental condition, likely because Al^3+^ is easily precipitated and does not easily penetrate into cells [86]. Together, these findings suggest that increased [Ca^2+^]_i_ enhances both Cu/Zn neurotoxicity and Zn neurotoxicity, whereas decreased [Ca^2+^]_i_ inhibits this neurotoxicity and that Ca^2+^ dyshomeostasis is critically involved in the mechanisms of both neurotoxicity.

The mechanism of Zn-induced elevations of [Ca^2+^]_i_ is of importance. Vander Jagt et al. also revealed that increased [Zn^2+^]_i_ causes Ca^2+^ increase in dendrites of CA1 pyramidal neurons [87]. Several studies have suggested the involvement of Zn in Ca homeostasis. Schulien et al. reported that Zn activates ryanodine-type Ca^2+^ channels [88]. Moreover, Zn reportedly regulates voltage-gated K^+^ channels [89], modulates neuronal excitability via several Ca^2+^ channels [90], and activates cation channels termed zinc-activated channels (ZACs) [91].

## 6. Hypothetical Schema of Cu/Zn Neurotoxicity

**Figure 4:**
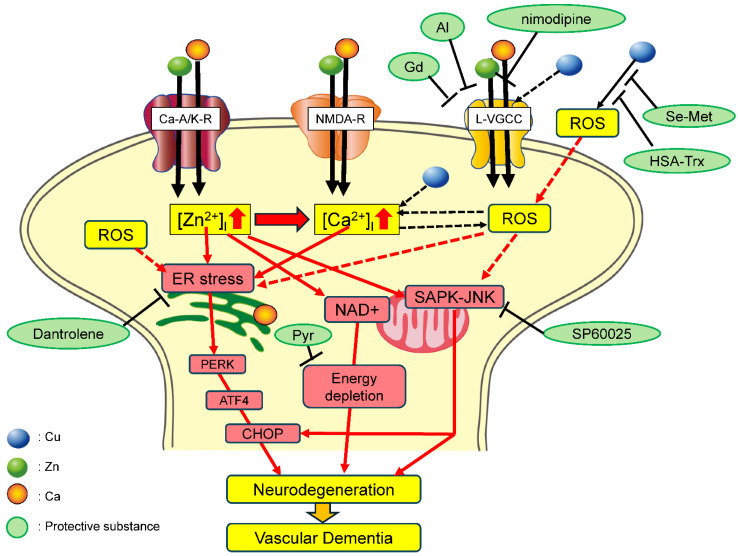
Hypothetical schema of the molecular pathways of Cu-enhanced Zn-induced neurotoxicity. Under pathological conditions, such as transient global ischemia and excess Zn and Cu, are secreted into the synaptic cleft. Zn and Ca are then intracellularly translocated via Ca-A/K-R, NMDA-R, and L-VGCC. Elevated [Zn^2+^]_i_ then triggers the ER stress pathway and stress-activated protein kinase/c-Jun amino-terminal kinase (SAPK/JNK) pathway, resulting in apoptotic neuronal death. Dantrolene, an inhibitor of the ER stress pathway, and SP600125, an inhibitor of the SAPK/JNK signaling pathway, attenuate Cu/Zn neurotoxicity. Zn also inhibits NAD^+^ and causes energy depletion in the mitochondria. Furthermore, Zn causes elevated [Ca^2+^]_i_, which then triggers various neurodegenerative processes. Substances that induce [Ca^2+^]_i_ elevation (e.g., KCl) enhance Cu/Zn neurotoxicity, whereas substances that inhibit [Ca^2+^]_i_ elevation (e.g., nimodipine, Al^3+^, and Gd^3+^) attenuate the neurotoxicity. It is also possible that Cu influences Zn-induced neurotoxicity because Cu produces reactive oxygen species (ROS), which upregulate the ER stress and SAPK/JNK pathways. Thus, the presence of Cu exacerbates Zn-induced neuronal death. Additionally, seleno-L-methionine (Se-Met) and a conjugated protein consisting of thioredoxin and human serum albumin (HSA-Trx) suppress ROS production and attenuate Cu/Zn neurotoxicity. It is also possible that Cu causes elevated [Ca^2+^]_i_ and enhances Zn-induced neurotoxicity. Ultimately, the coexistence of Cu and Zn in the same synapse triggers neurodegenerative pathways and eventually induces the pathogenesis of vascular-type senile dementia. Colored circles represent Zn, Cu, and Ca.

On the basis of these results, we propose a hypothetical schema regarding the molecular pathways of Cu/Zn neurotoxicity (Figure 4). At least five pathways are possibly involved in Cu/Zn neurotoxicity: ER stress pathway, SAPK/JNK pathway, mitochondrial energy failure, ROS production, and Ca homeostasis disruption.

After exposure to Zn, Zn^2+^ is translocated via Ca-A/K-R, NMDA-R, and L-VGCC alongside Ca^2+^. Increased [Zn^2+^]_i_ then triggers the ER stress and SAPK/JNK signaling pathways, which lead to several apoptotic pathways. Dantrolene, an inhibitor of ER stress, and SP600125, an inhibitor of the SAPK/JNK signaling pathway, can attenuate Cu/Zn neurotoxicity. Zn also inhibits NAD^+^ and causes mitochondrial injury and energy depletion. Pyruvate (Pyr) affects these pathways and attenuates neurotoxicity.

Furthermore, Zn causes increased [Ca^2+^]_i_, which then triggers numerous neurodegenerative pathways. Exposure to high concentrations of KCl, which causes neuronal depolarization and increases [Ca^2+^]_i_, enhances both Cu/Zn neurotoxicity and Zn neurotoxicity. Ca^2+^ channel inhibitors, including nimodipine, Al^3+^, and Gd^3+^, can attenuate the neurotoxicity by blocking the Zn-induced [Ca^2+^]_i_ rise.

There are several possibilities about the roles of Cu in Zn-induced neurotoxicity. As noted, exposure to Cu produces ROS. It is widely known that ROS induces the ER stress pathway [92] and SAPK/JNK pathways [93]. Therefore, Cu exacerbates Zn neurotoxicity via the induction of these neurodegenerative pathways. Antioxidants such as HSA-Trx and seleno L-methionine (Se-Met) can inhibit ROS production and attenuate Cu/Zn neurotoxicity. It is also possible that Cu may influence Ca dyshomeostasis. Cu reportedly enhances neural excitability [94]. Both Zn and Cu reportedly cause Ca release from the sarcoplasmic reticulum [95]. Moreover, there are close connections between oxidative stress and Ca^2+^ homeostasis. Li et al. demonstrated that Cu-induced ROS causes ER stress via an increase in [Ca^2+^]_i_ [96]. The elevations of [Ca^2+^]_i_ possibly cause the production of ROS and vice versa and then trigger neurodegenerative processes. Therefore, Zn and Cu may collaborate to induce neuronal death after ischemia via these neurodegenerative pathways, ultimately contributing to the pathogenesis of VTD. It is also possible that the disruption of Zn, Cu, and/or Ca homeostasis may trigger other neurodegenerative diseases such as prion diseases and Alzheimer’s disease [51].

Protective substances against Cu/Zn neurotoxicity may suppress neurodegeneration after transient global ischemia and may lead to the development of drugs for the prevention or treatment of VTD. We focused on carnosine [19] and Se-Met [97] as endogenous neuroprotectors. Moreover, substances that regulate Ca homeostasis will provide good candidates for such drugs. Further research about the mechanisms underlying the Zn-induced [Ca^2+^]_i_ rise is needed.

## 7. Conclusions

In the present review, we have described the collaborative effects of Cu in Zn-induced neuronal death and have summarized the possible contributions of Cu and Zn in the pathogenesis of VTD, with a focus on Ca homeostasis. In the synapse, these three elements (Zn, Cu, and Ca) are abundant and crosstalk with each other via disease-related proteins. It is therefore possible that the disrupted homeostasis of Zn, Cu, and Ca may trigger VTD and other neurodegenerative diseases. Our working hypothesis of the molecular mechanisms related to Cu/Zn neurotoxicity may aid in both the understanding of VTD pathogenesis and in the development of drugs for the treatment and/or prevention of VTD.

## Data Availability

Not applicable.

## Abbreviations

APP: amyloid precursor protein, ATF4: activating transcription factor 4, ATP7A: copper-transporting ATPase 7A, AMPA: α-amino-3-hydroxy-5-methylisoxazole-4-propionic acid, Arc: activity-related cytoskeleton protein, Ca EDTA: Ca ethylenediaminetetraacetic acid, CSF: cerebrospinal fluid, CHOP: CCAAT-enhancer-binding protein homologous protein, Ctr1: copper transporter 1, D-APV: 2-amino-5-phosphonovalerate, ER: endoplasmic reticulum, GABA: γ-aminobutyric acid, GADD34: growth arrest and DNA-damage-inducible gene 34, IRE1: inositol requiring enzyme 1, NMDA: N-methyl-D-aspartate, MT: metallothionein, PERK: PKR-like endoplasmic reticulum eIF2a kinase, PrP: prion protein, ROS: reactive oxygen species, RT-PCR: reverse transcription polymerase chain reaction, SAPK/JNK: stress-activated protein kinases/c-Jun amino-terminal kinases, Se-Met: seleno L methionine, VTD: vascular type dementia, VGCC: voltage-gated Ca^2+^ channel, [Zn^2+^]_i_: intracellular level of Zn^2+^.
